# Interleukin-18 alters protein expressions of neurodegenerative diseases-linked proteins in human SH-SY5Y neuron-like cells

**DOI:** 10.3389/fncel.2014.00214

**Published:** 2014-08-07

**Authors:** Elina M. Sutinen, Minna A. Korolainen, Jukka Häyrinen, Irina Alafuzoff, Steven Petratos, Antero Salminen, Hilkka Soininen, Tuula Pirttilä, Johanna O. Ojala

**Affiliations:** ^1^Department of Neurology, Institute of Clinical Medicine, University of Eastern FinlandKuopio, Finland; ^2^Brain Research Unit, Clinical Research Centre, University of Eastern FinlandKuopio, Finland; ^3^Institute of Biotechnology, University of HelsinkiHelsinki, Finland; ^4^School of Medicine, Institute of Biomedicine, University of Eastern FinlandKuopio, Finland; ^5^Rudbecklaboratoriet, Department of Immunology, Genetics and Pathology, Molecular and Morphological Pathology, Uppsala UniversityUppsala, Sweden; ^6^Regenerative Neuroscience and Development Laboratory, Department of Medicine, Central Clinical School, Monash UniversityPrahran, VIC, Australia; ^7^Department of Neurology, Kuopio University HospitalKuopio, Finland

**Keywords:** Interleukin-18, inflammation, neurodegenerative diseases, proteomics, oxidative stress related proteins, CRMP2, DDAH, MMP14

## Abstract

Chronic inflammation and oxidative stress (OS) are present in Alzheimer's disease (AD) brains in addition to neuronal loss, Amyloid-β (Aβ) plaques and hyperphosphorylated tau-protein neurofibrillary tangles (NFTs). Previously we showed that levels of the pro-inflammatory cytokine, interleukin-18 (IL-18), are elevated in post-mortem AD brains. IL-18 can modulate the tau kinases, Cdk5 and GSK3β, as well as Aβ-production. IL-18 levels are also increased in AD risk diseases, including type-2 diabetes and obesity. Here, we explored other IL-18 regulated proteins in neuron-like SH-SY5Y cells. Differentiated SH-SY5Y cells, incubated with IL-18 for 24, 48, or 72 h, were analyzed by two-dimensional gel electrophoresis (2D-DIGE). Specific altered protein spots were chosen and identified with mass spectrometry (MS) and verified by western immunoblotting (WIB). IL-18 had time-dependent effects on the SH-SY5Y proteome, modulating numerous protein levels/modifications. We concentrated on those related to OS (DDAH2, peroxiredoxins 2, 3, and 6, DJ-1, BLVRA), Aβ-degradation (MMP14, TIMP2), Aβ-aggregation (Septin-2), and modifications of axon growth and guidance associated, collapsin response mediator protein 2 (CRMP2). IL-18 significantly increased antioxidative enzymes, indicative of OS, and altered levels of glycolytic α- and γ-enolase and multifunctional 14-3-3γ and -ε, commonly affected in neurodegenerative diseases. MMP14, TIMP2, α-enolase and 14-3-3ε, indirectly involved in Aβ metabolism, as well as Septin-2 showed changes that increase Aβ levels. Increased 14-3-3γ may contribute to GSK3β driven tau hyperphosphorylation and CRMP2 Thr514 and Ser522 phosphorylation with the Thr555-site, a target for Rho kinase, showing time-dependent changes. IL-18 also increased caspase-1 levels and vacuolization of the cells. Although our SH-SY5Y cells were not aged, as neurons in AD, our work suggests that heightened or prolonged IL-18 levels can drive protein changes of known relevance to AD pathogenesis.

## Introduction

The most common form of dementia is sporadic, late-onset Alzheimer's disease (AD), the etiology of which is still unknown. As well as contributory environmental factors and increased incidence in females, well-established risk factors for sporadic AD include aging, type-2 diabetes and obesity, which all involve increased inflammation levels (Bendlin et al., 2010; Hamer et al., 2012). The neuropathological hallmarks of AD include extracellular neuritic amyloid-β (Aβ) plaques and intraneuronal neurofibrillary tangles (NFTs) containing hyperphosphorylated tau (Braak and Braak, 1997). In addition to neuronal loss, chronic inflammation, oxidative stress (OS), and excitotoxic damage characterize AD brains (Braak and Braak, 1991; von Bernhardi and Eugenín, 2012; Morales et al., 2014). However, the pathological sequelae and the interaction of these mechanisms during the course of the disease are largely unknown.

Inflammation in the brain is primarily driven by activated microglia and astrocytes, leading to the over-production of inflammatory cytokines and other inflammatory products in association with increased reactive oxygen species (ROS) (Morales et al., 2014). The interleukin-1 family member interleukin-18 (IL-18), also known as IL-1γ or interferon-γ-inducing factor (IGIF), is an inflammatory cytokine that is produced mainly by activated microglia in the brain. Previously we showed that expression of this upstream cytokine is elevated in many AD brain regions (Ojala et al., 2009). IL-18, indirectly via interferon-γ (IFNγ) induction, can also increase indoleamine 2,3-dioxygenase (IDO), in turn producing neuroregulatory and excitotoxic tryptophan catabolites that are proposed to contribute to AD neuropathology (Guillemin et al., 2005; Anderson and Ojala, 2010). As with IL-1β, IL-18 is cleaved by caspase-1 [interleukin-1β converting enzyme (ICE)] to an active secreted form. In comparison to IL-1β, IL-18 expression is higher, partly due to its longer half-life (Ojala et al., 2009), suggesting that it may exert a more significant impact in the brain than the more extensively researched IL-1β. The normal function of brain IL-18 is unknown, but it may have a role in central nervous system development as well as contributing to brain immune and inflammatory processes (Ojala et al., 2008; Alboni et al., 2010). Interestingly, increased IL-18 levels have also been detected in type-2 diabetes (Aso et al., 2003), obesity (Esposito et al., 2002), and ischemic heart disease (Mallat et al., 2001). IL-18 is also induced during physical/emotional stress responses (Sugama and Conti, 2008; Yaguchi et al., 2010), and is a susceptibility factor for depression (Haastrup et al., 2012), another condition that is intimately associated with neurodegenerative processes (Diniz et al., 2013).

Here we tested time-related impacts of pro-inflammatory IL-18 in pure human neuron-like cells. We hypothesized that IL-18 has a role in the development of neuropathological changes related to several neurodegenerative diseases including AD. This is based on our recent findings showing IL-18 effects on tau and its phosphorylation (Ojala et al., 2008), as well as on amyloid precursor protein (APP) processing and Aβ production (Sutinen et al., 2012) in differentiated SH-SY5Y neuron-like cells. The main aim of the present study was to discover other relevant molecules and/or mechanisms in these human cells that are regulated by IL-18 and that may have general importance in the pathogenesis of neurodegenerative diseases, including AD. We used two-dimensional difference-in-gel electrophoresis (2D-DIGE) and mass spectrometry (MS) to find IL-18 regulated targets. The findings were verified by western immunoblotting (WIB).

Our study showed that IL-18 induces time-dependent effects on protein expression profiles in differentiated neuron-like SH-SY5Y cells. The most significant protein changes were found at 24 h but were also evident at 48 and 72 h post-IL-18 treatment, vs. untreated control cells. Following the detection of altered proteins, we focused on targets related to OS. The most interesting targets, DDAH, Septin-2 and MMP14 were also studied from frontal and occipital lobes of AD-patients and healthy controls, as well as the impact of Aβ on their expression. We also evaluated the vacuolization of the neuron-like SH-SY5Y cells and their caspase-1 expression.

## Materials and methods

### Cell culture and treatments

SH-SY5Y neuroblastoma cells (DSMZ, Germany) were cultured in Dulbeccos's medium (BioWhittaker/Cambrex, Belgium) containing 4.5 g/L glucose, 5% fetal bovine serum (FBS; HyClone/Pierce; Logan, UT, USA), 2 mM L-glutamine (Cambrex/Lonza), 100 U/ml penicillin and 10 μg/mL streptomycin (Cambrex/Lonza) in a humidified atmosphere 5% CO_2_ at 37°C. The cells were plated as 3 × 10^5^ cells/well into the 6-well plate (Nunc™, Denmark), and differentiated within 3 days of treatment with 10 μM all-trans retinoic acid (ATRA; Sigma-Aldrich, St. Louis, MO, USA) followed by 4 days of 50 ng/mL human recombinant brain derived neurotrophic factor (BDNF, Alomone labs, Israel) (Sutinen et al., 2012). Bioactive recombinant human IL-18 (MBL Medical Biological laboratories Co, Ltd., Japan) was added to the culture medium at 150 ng/mL for 24, 48, and 72 h. BDNF was also present during the IL-18 treatments. Since the concentration of IL-18 and BDNF is not known in the very close proximity of the release site, we used an excess concentration (physiological 40–80 ng/ml), also due to their recombinant origin and effort to force the time frame of the experiment to 72 h due to the technical reasons. Also IL-18 binding protein (IL-18BPa/Fc Chimera; R&D Systems) and Aβ42 (Bachem) were used in some experiments.

After the treatment, the washed cells were lysed by using M-PER^®^ Mammalian Protein Extraction Reagent (Pierce). The culture medium was also collected. The protein content was determined with DC Protein assay (BioRad) and samples were stored in −80°C.

### Protein labeling

Protein samples (135 μg) were prepared from lysates of control and IL-18 treated cells, then purified using the 2-D clean up-kit (GE Healthcare) in accordance with the manufacturer's protocol. Samples of culture medium from the controls and IL-18 treated (500 μL) wells were concentrated to 15 μL by using Nanosep 3K omega centrifugal devices (Pall Corporation). CyDye DIGE Fluor, minimal labeling kit (GE Healthcare) was used for labeling of the lysate or medium proteins. Internal standards, consisting of an aliquot of all the samples to be used in a particular experiment, was mixed and labeled with Cy2 dye. Control and IL-18 treated samples were randomized and half of these samples were labeled with Cy3 and the other half with Cy5. All dyes were incubated for 30 min in the dark on ice in accordance with the manufacturer's protocol (GE Healthcare). Labeling was terminated by incubation with 1 μL of 10 mM L-lysine for 10 min on ice.

### Two-dimensional gel electrophoresis (2D-DIGE)

The first dimension 4–7 NL ImmobilineTM DryStrips, 24 cm (GE Healthcare) were rehydrated over night by using DeStreakTM rehydration solution (GE Healthcare) and IPG buffer pH 4–7 (GE Healthcare). The labeled samples were formulated for the strips by adding an equal volume of 2× buffer consisting of 7 M urea, 2 M thiourea, 4% 3-[(3-cholamidopropyl)dimethylammonio]-1-propanesulphonate (CHAPS), 2% IPG buffer and 2% dithiothreitol (DTT) (GE Healthcare), followed by incubation on ice for 15 min, after which the samples were cup-loaded into the rehydrated strips. The first dimension was run by applying 500 V (500 kVh), 1000 V (5200 kVh), 8000 V (16500 kVh), and finally 8000 V (42200 kVh) by using an EttanTM IPGphor unit (Amersham). The focused strips were equilibrated in 2% sodium dodecyl sulfate (SDS) equilibration buffer containing 6 M urea, 50 mM Tris pH 8.8, 30% glyserin and 0.002% bromophenol blue, first with added 1% DTT at RT for 12 min, followed by buffer supplemented with 2.5% iodoacetamide (IAA) for another 12 min at room temperature (RT). After the equilibration, the strips were loaded into the 12.5% SDS-polyacrylamide gels (1 mm) in an Ettan DALT-separation unit (Amersham) and run at 12 W/gel for 30 min and 128 W/gel until the bromophenol blue front reached the bottom of the gel. Each gel run contained six separate experiments from the same time-point. Since the minimal labeling technique (Unlü et al., 1997) labels each protein with a maximum of only one dye molecule per protein (Swatton et al., 2004) and all of the dyes have identical molecular weights (MW) and isoelectric points (pI) but differ in their excitation and emission wavelengths, the MW of the proteins were minimally affected. The 2D images were visualized by Typhoon 9400 Variable Mode Imager scanner (GE Healthcare) using 488, 523, and 633 nm lasers for Cy2, Cy3, and Cy5 dyes.

### Spot analysis

All the protein-spots in the gel images were quantified by using the DeCyder 6.5.11 software (GE Healthcare). A DeCyder differential in-gel analysis module was used for pair wise comparisons of each sample (Cy3 and Cy5) to the Cy2 mixed standard present in each gel. The DeCyder biological variation analysis module was then used to simultaneously match all the protein-spot images from the six gels and to calculate average abundance ratios across the samples. The protein-spots in the gels were compared to the 2D map databases (SWISS-2DPAGE and SWISS-PROT).

### Mass spectrometry (MS)

Differentiated proteins within the gels were stained with PlusOne™ Silver Staining Kit (GE Healthcare) according to manufacturer's instructions. The proteins of interest were cut out manually from the silver stained gels, shrank with acetonitrile (ACN) and cleaved with trypsin (Sequencing grade Modified Trypsin V5111; Promega, Madison, WI, USA) at +37°C overnight. Protein MS identification was done in collaboration with the protein chemistry research facility in University of Helsinki, Viikki, and Department of Biosciences, University of Eastern Finland, Kuopio. In the University of Helsinki, the cleaved samples were purified with ZipTip-method and eluted directly from the tips onto a MTP 384 ground steel sample plate with saturated α-cyano-4-hyrdoxycinnamic acid in 0.1% trifluoroacetic acid (TFA) and 60% ACN. Matrix-assisted laser desorption ionization—time of flight (MALDI-TOF) mass spectrometer mass spectra for peptide mass fingerprints (PMF) and MALDI-TOF/TOF mass spectra for identification by fragment ion analysis were acquired in positive ion reflectron mode using an Ultraflex TOF/TOF instrument (Bruker Daltonik GmbH, Bremen, Germany). The PMF spectra were processed with FlexAnalysis (version 3.0) and spectra were internally calibrated by using trypsin peaks. Separation of the protein digests liquid chromatography-MS/MS (LC-MS/MS) were done on a C18 column by using a nanoscale high-performance liquid chromatography (HPLC) system (Ultimate 3000; Dionex, Sunnyvale, CA). The samples were concentrated and desalted on a C18 precolumn (PROTECOL; SGE Analytical Science, Griesheim, Germany) at a flow rate 7 μL/min (0.1% TFA). Peptides were eluted with a linear gradient of ACN in 0.1% formic acid (FA) at a flow rate of 200 nL/min. MS/MS of tryptic peptides was conducted on a hybrid quadrupole/TOF mass spectrometer with NanosprayII source (QSTAR Elite; Applied Biosystems, Foster city, CA). The Nanospray was generated using a PicoTip needle. The peak list was generated with ProteinPilot-software (Applied Biosystem).

At the University of Eastern Finland, the tryptic peptides were separated using the Ultimate/Famos/Switchos capillary liquid chromatography system (LC Packings, Amsterdam, Netherlands). The samples were filtered on-line through PEEK encapsulated titanium filter (VICI/ Valco, Houston, TX, USA) and trapped onto PepMap 100 C18 pre-column (Dionex, Sunnyvale, CA, USA) in 0.1% FA (Sigma Aldrich, Steinhelm, Germany) with 2% ACN (Sigma Aldrich) at a flow rate of 30 μL/min using an Applied Biosystems 400 Solvent Delivery System (Applied Biosystems). After the 3 min of loading and clean-up, the pre-column was automatically switched in-line with a C18 Mass Spec analytical column (Grace Vydac, Hesperia, CA, USA). The peptides were eluted using a linear ACN gradient starting from 0.1% FA with 5% ACN to 30% of 0.1% FA with 95% ACN in 35 min, and finally to 100% of 0.1% FA with 95% ACN. The LC flow was connected to the mass spectrometer by a nano-electrospray ionization MS (ESI) ion source (MDS Sciex, South San Francisco, CA, USA) using distally coated 10 μm PicoTip emitters (New Objective, MS Wil GmbH, Switzerland). Mass spectra were recorded in a positive mode on a QSTAR XL hybrid quadrupole TOF instrument (Applied Biosystems), using information-dependent acquisition for obtaining MS/MS data.

### Protein identification

Database searches with obtained data were performed with Mascot search engine against the SwissProt database (http://www.matrixscience.com). The search criteria for both Mascot searches were human-specific taxonomy, trypsin digestion with one missed cleavage allowed, carbamidomethyl modification of cysteine as a fixed modification and oxidation of methionine as a variable modification. For the PMF spectra the maximum peptide mass tolerance was ±50 ppm. For the LC-MS/MS spectra both the maximum precursor ion mass tolerance and MS/MS fragment ion mass tolerance were 0.2 Da and peptide charge state of +2 or +3 was used.

### Human samples

DDAH2, SEPT2 and MMP14 were also examined from post-mortem human brain samples (occipital and frontal lobes as pairs from the same individual). Control samples included 3 women and 3 men (age 83.3 years, SD ±5.7; Braak 1.17 ±0.75, obduction delay 5.7 h ± 2.5; cause of death: four cardiac problems, one pneumonia, one pulmonary embolism). The AD patients included 7 women and 2 men (age 83.1 years, SD ±8.1; Braak 5.22 ±0.67, obduction delay 5.3 h ± 3.0; cause of death: five pneumonia, two cardiac reasons, two pulmonary embolisms). The samples were stored at −80°C until used. The brain tissue samples were homogenized on ice into the lysis buffer containing 10 mM Tris pH 8.0, 150 mM NaCl, 1% NP-40, 1% Triton X-100, 2 mM EDTA, and Complete Protease inhibitors (Roche Diagnostics; Mannheim, Germany) using a pellet pestle (Kontes; Vineland, NJ, USA). Protein levels were estimated with the DC protein assay kit (Bio-Rad Laboratories; Hercules, CA, USA) according to manufacturer's protocol.

The study followed the recommendations for biomedical research involving humans (Declaration of Helsinki of The World Medical Association 1964 including the revisions and clarifications up to Tokyo 2004 and CIOMS international ethical guidelines for biomedical research involving human subjects), as well as the law concerning information protection. Further, the human studies were approved by the Ethics Committee of Kuopio University Hospital. The written informed consents were obtained from the subjects or their representatives by the clinicians at the Department of Neurology, Kuopio University Hospital. Permission to obtain the post-mortem tissue was provided by the National Board of Medical Legal Affairs/National Supervisory Authority for Welfare and Health.

### Western immunoblotting (WIB)

For WIB, equal amounts of protein (25 or 35 μg) from cell lysates or human brain samples, and equal volumes of cell culture media (120 μL) were electrophoresed in 12.5% SDS-polyacrylamide gels. Proteins were transferred to Hybond-P PVDF membrane (Amersham Biosciences/GE Healthcare). Full-Range and Low-Range Rainbow Molecular Weight Markers (GE Healthcare) as well as Spectra™ Multicolor Broad Range Protein Ladder (Fermentas) were used for the detection of target proteins correct MW. The antibodies were commercial and evaluated by the suppliers and end users (Table 1). However, we performed some characterization studies for a number of antibodies that required validation due to a partially limited use of some of them in the literature. Those studies included different cell lines known to express or had low expression of the targets as well as known inducers (Supplementary data 1). A peptide inhibition assays were also performed to determine the specificity of CRMP2 Thr555 (Supplementary data 2). The membranes were blocked with 5% non-fat milk (Valio, Finland) in phosphate buffered saline (PBS) containing 0.05% Tween-20 (Fluka/Sigma-Aldrich; Steinheim, Germany) or alternatively in Tris buffered saline (TBS) containing 0.1% Tween-20 for 1–2 h. The milk-blocked membranes were immune detected for 1–2 h at RT or over night at +4°C with selected primary antibodies (diluted in 1:400), and washed with PBS/0.05% Tween-20 or TBS/0.1% Tween-20 for 3 × 5 min. The primary antibodies are collected in Table 1. The antibodies were purchased from Santa Cruz Biotechnology, Inc. (Santa Cruz, CA, USA), ECM Biosciences, Abcam (Cambridge, UK), Millipore, Sigma-Aldrich and GE Healthcare. The secondary antibodies were used as 1:10000 dilution and 1.5–2 h incubation time.

**Table 1:** **The list of antibodies used in the study**.

	**Provider**	**Cat N:o**	**Raised in**	**Purification**	**Sequence**	**Certification for specificity (company), user, our studies**
**PRIMARY ANTIBODY**						
PRX2 (N-13)	Santa Cruz Bio.	sc-23967	goat pc	affinity pur.	h, near the N-terminus	(WB); Petrak et al., 2007; suppl.1
PRX3 (12B)	Santa Cruz Bio.	sc-59661	mouse mc		h, full length PRX3	(WB, IF); McCommis et al., 2011; suppl.1
PRX6 (E-18)	Santa Cruz Bio.	sc-55013	goat pc	affinity pur.	h, internal region	(WB); suppl.1
DJ-1 (N-20)	Santa Cruz Bio.	sc-27004	goat pc	affinity pur.	h, near N-terminus	(WB, IF); Batelli et al., 2008; suppl.1
BLVRA (2E4)	Santa Cruz Bio.	sc-100511	mouse mc		h, recombinant BLVRA	(WB); suppl.1
DDAH2 (C-19)	Santa Cruz Bio.	sc-26071	goat pc	affinity pur.	h, C-terminus	(WB); Park et al., 2008; suppl.1
TIMP2 (H-140)	Santa Cruz Bio.	sc-5539	rabbit pc		h, aa 81-220	(WB, IHC); Polyakova et al., 2008; suppl.1
MT-MMP-1 (H-72)	Santa Cruz Bio.	sc-30074	rabbit pc		h, aa 511-582	(WB, IHC); Saygili et al., 2011; suppl.1
SEPT2 (N-12)	Santa Cruz Bio.	sc-20408	goat pc	affinity pur.	h, near the N-terminus	(WB, IHC); Maimaitiyiming et al., 2008; suppl.1
α Enolase (L-27)	Santa Cruz Bio.	sc-100812	mouse mc		h, recombinant α-enolase	(WB, IF, IHC); Yu et al., 2010; suppl.1
γ Enolase (N-14)	Santa Cruz Bio.	sc-31859	goat pc	affinity pur.	h, near N-terminus	(WB), Yamamoto et al., 2011; suppl.1
14-3-3-γ (C-16)	Santa Cruz Bio.	sc-731	rabbit pc	affinity pur.	h, C-terminus	(WB, IF, IHC); Schindler et al., 2006; Shiga et al., 2006; suppl.1
14-3-3-ε (E-20)	Santa Cruz Bio.	sc-31962	goat pc	affinity pur.	h, internal region	(WB); suppl.1
CRMP2 (clone 1B1)	Santa Cruz Bio.	sc-101348	mouse mc		h, synthetic peptide derived from the C-terminus	(WB); Chi et al., 2009; suppl.1
CRMP2 (Ser522), phospho-specific	ECM Biosci.	CP2191	rabbit pc	affinity pur.	h, P-CRMP (S522) synthetic peptide (coupled to carrier protein) corresponding to aa:s surrounding S522	(WB); Yoneda et al., 2012
CRMP2 (Thr555), phospho-specific	ECM Biosci.	CP2251	rabbit pc	affinity pur.	h, P-CRMP2 (T555) synthetic peptide (couple to carrier protein) corresponding to aa:s surrounding T555	(WB, ICC); suppl.2
CRMP2 (Thr514)	Abcam	ab62478	rabbit pc	affinity pur.	h, synthetic phosphopeptide derived from the phosphorylation site of T514 (T-V-T^p^-P-A)	(WB, ICC, IHC), Lim et al., 2014
Caspase 1 (14F468)	Santa Cruz Bio.	sc-56036	mouse mc		h, aa 371-390	(WB); Zhang et al., 2012; suppl.1
Anti-Aβ [4-10]-antibody (W0-2)	Millipore	MABN10	mouse mc	protein G pur.	h, amyloid β-peptide	(WB, IHC); Ida et al., 1996
Anti-Alzheimer precursor protein A4 (22C11)	Millipore	MAB348	mouse mc	purified Ig	pur. rc Alzheimer preucrsor A4 (pre4695) fusion protein	(WB, IHC); Mudher et al., 2001
Actin (H-196)	Santa Cruz Bio.	sc-7210	rabbit pc		h, aa 180-375	(WB, intracell. FCM anal.); Calegari et al., 2011
α-Tubulin (B-5-1-2, mouse ascites fluid)	Sigma-Aldrich	T 5168	mouse mc		sarkosyl resistant filaments from sea urchin sperm axonemes	(WB, ICC); Czymai et al., 2010
**SECONDARY ANTIBODY**						
anti-rabbit	GE Healthcare	NA9340	donkey	affinity pur.	rabbit IgG, peroxidase-linked species specific (Fab')_2_ fragment	(WB, IHC)
anti-mouse	GE Healthcare	NA931	sheep	affinity pur.	mouseit IgG, peroxidase-linked species specific (whole antibody)	(WB, IHC)
anti-goat IgG, (Fab')_2_-HRP	Santa Cruz Bio.	sc-3851	donkey	affinity pur.	goat IgG, conjugated with HRP	(WB)

The references are listed in supplementary data.

h, human; mc, monoclonal; pc, polyclonal, suppl., supplementary data.

pur., purified; Ig, immunoglobulin; aa, amino acid; rc, recombinant; HRP, horse radish peroxidase.

WB, western immunoblot; ICC, immunocytochemistry; IF, immunofluorescence; IHC, immunohistochemistry; FCM, flow cytometry.

After the incubations, the membranes were washed as described for 4 × 5 min followed by 15 min wash with PBS or TBS. The targets were detected with Immobilon Western Chemiluminescent HRP Substrate (Millipore; Billerica, MA, USA) and documented to Fujifilm Super RX (Fuji Photo Film Co., Ltd.). The x-ray films were scanned and analyzed with MCID M5-image analysis system (Imaging Research Inc.; Ontario, Canada). The same membrane was used in three to four different antibody reactions after stripping with four times wash with TBS/0.1% Tween-20 at RT followed by shaking in stripping buffer (62.7 mM Tris pH 6.8, 2% SDS, 0.7% β-mercaptoethanol) at +50°C for 30 min and washing six times with TBS/0.1% Tween-20 at RT.

### Statistics

The separate experiments and samples (*n* = 5–17) were analyzed with parametric paired Student's *t*-test (T-t) and non-parametric Mann–Whitney *U*-test (M-W).

## Results

### 2D-DIGE analysis of differentiated SH-SY5Y neuron-like cells treated with IL-18

#### IL-18 altered protein profiles in SH-SY5Y cells in time related manner

**Figure 1:**
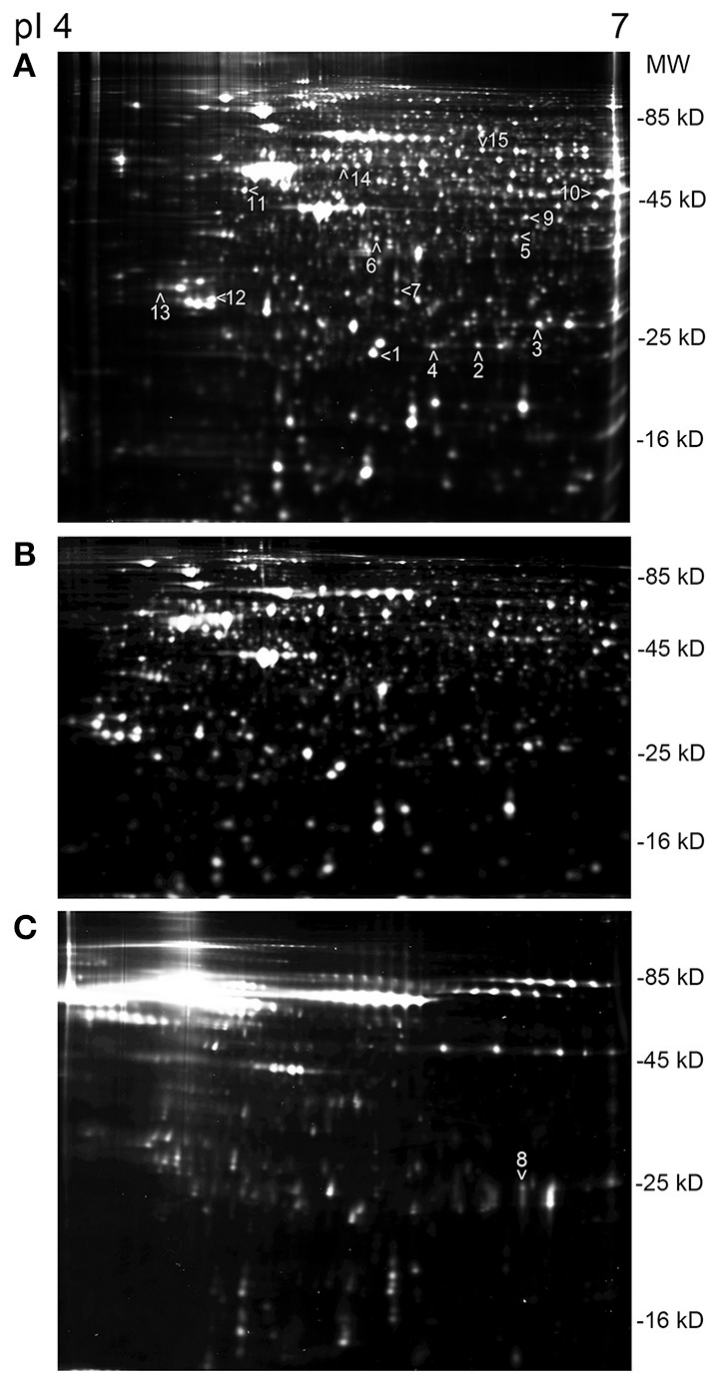
**An example sets of 2D-DIGE protein profiles of SH-SY5Y cells. (A)** Differentiated SH-SY5Y cells, at the 48 h time-point where the number of protein spots was the greatest. The arrow heads indicate the picked proteins, done from the silver stained gels; protein ID: (1) PRX2, (2) PRX3, (3) PRX6, (4) DJ-1, (5) BLVRA, (6) DDAH1, (7) DDAH2, (8) TIMP2, (9) SEPT2, (10) ENOA, (11) ENOG, (12) 14-3-3γ, (13) 14-3-3ε, (14) RUFY3, (15) CRMP2. **(B)** Undifferentiated SH-SY5Y neuroblastoma cells showed the least number of spots, in comparison to differentiated SH-SY5Y cells in **(A)**. **(C)** Protein profile of the culture medium of the differentiated SH-SY5Y cells at the 72 h time-point. Protein ID 8, TIMP2, was picked from the medium gel. The strip pI range was the same in all shown gels.

**Figure 2:**
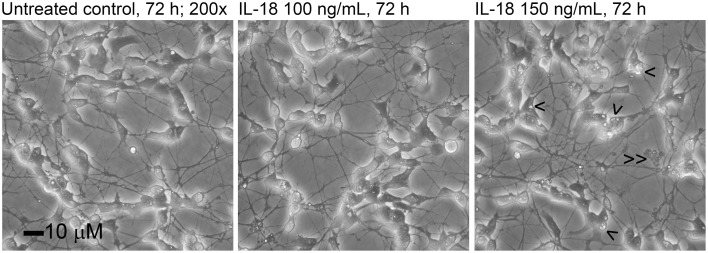
**The appearance of the differentiated SH-SY5Y cells treated with 100 or 150 ng/ml of IL-18 for 72 h**. The number of vacuoles (>) was higher in 150 ng/ml treated cells (about 4.1/ cell) than in untreated control (2.4/ cell) or 100 ng/ml treated cells (3.5/ cell). Some cell debris is also apparent (> >).

Time-dependent changes in the protein levels were apparent in differentiated SH-SY5Y cells after 150 ng/mL IL-18 treatments for 24, 48, or 72 h, compared to respective untreated control cells (Table 2). The altered protein profiles with greatest differences were identified in the cells exposed to IL-18 for 24 h, approximately 4.2% of the total protein spots were differentially expressed. From this total of 81 differentially expressed proteins, 27 proteins were up- and 54 down-regulated by IL-18 treatment. IL-18 addition for 48 and 72 h also showed significant protein changes vs. untreated cells (48 h: total 65; 45 increased, 20 decreased, approximately 3.3%; 72 h: total 51; 31 increased, 20 decreased, approximately 2.7%). The number of protein spots was greatest at the 48 h time-point (1965 spots) (Figure 1A), suggesting enhanced translational modifications occurring in early expressed proteins in comparison to later proteins. Total protein spot number was lowest in non-differentiated, untreated SH-SY5Y neuroblastoma cells, being 1754 (Figure 1B). IL-18 also induced some changes in the culture medium at the 72 h time-point (Figure 1C). However, the separation of secreted proteins from cell debris of the closed cell culture system proved difficult. Although the culture medium was taken gently from the cultures and centrifuged prior to use, the medium likely contained some ruptured cell contents due the delicate nature of the neurites. Therefore, due to the great variation in the number of protein spots, the medium was not possible to analyse in detail with this number of experiment repeats (*n* = 6). Nevertheless, the appearance of the IL-18 treated cells at the 72 h time-point prior to harvest is shown in Figure 2. The 150 ng/ml IL-18 treated cells were more vacuolized [4.1 vacuoles/ cell, standard error of mean (s.e.m.) ± 0.17; *n* = 90; *p* ≤ 0.000 (T-t, M-W)] as well as 100 ng/ml treated cells [3.5 ±0.15; *n* = 90; *p* ≤ 0.000 (T-t, M-W)] compared to untreated control cells (2.4 ± 0.12; *n* = 90). When 150 ng ml/ IL-18 treated cells were compared to 100 ng/ml treated ones, the increase of vacuole number was about 15.1% [*p* = 0.019 (T-t); *p* = 0.026 (M-W)]. Cell debris was also apparent indicating apoptotic cells (Figure 2).

**Table 2:** **IL-18 treatment induced protein changes in differentiated SH-SY5Y cell lysates, including alteration in levels and modifications, vs. to untreated control at different time-points (DeCyder spot detection-software)**.

**Time-point**	**Changed protein spots**	**Up-regulated**	**Down-regulated**	**Total number of spots**
24 h	81 (~4.2%)	27	54	1934
48 h	65 (~3.3%)	45	20	1965
72 h	51 (~2.7%)	31	20	1866

Some of the proteins exhibiting change under 2D-DIGE were selected (Figures 1A,C) and identified by MS. From the 79 identified proteins, 29% were involved in proliferation/ differentiation, about 18% in cell signaling, 16% in transport and 13% in the regulation of oxidation. Proteins of interest were selected as shown in Table 3. From these proteins, 14 were of known relevance to neurodegenerative diseases and so were further analyzed by WIB.

**Table 3:** **Proteins of human SH-SY5Y cells, identified with mass spectrometry and studied further with western immunoblots**.

**Id no**	**Protein name**	**Gene name**	**SwissProt**	**ID type**	**No. of peptides**	**Sequence coverage (%)**	**Mowse score**	**MW**	**pI**
1	Peroxiredoxin-2	PRDX2_HUMAN	P32119	ESI	4	19	87	21892	5.67
2	Thioredoxin-dependent peroxidase reductase	PRDX3_HUMAN	P30048	MALDI	7	44	57	27693	5.77
3	Peroxiredoxin-6	PRDX6_HUMAN	P30041	MALDI	23	83	272	25035	6.00
4	Protein DJ-1 (Park 7)	PARK7_HUMAN	Q99497	ESI	7	38	149	19891	6.32
5	Biliverdin reductase A	BIEA_HUMAN	P53004	MALDI	14	41	159	33428	6.06
6	N(G), N(G)-dimethylarginine dimethylaminohydrolase	DDAH1_HUMAN	O94760	MALDI	30	66	235	31122	5.53
7	N(G), N(G)-dimethylarginine dimethylaminohydrolase 2	DDAH2_HUMAN	O95865	MALDI	7	39	96	29644	5.66
8	Metalloproteinase inhibitor 2	TIMP2_HUMAN	P16035	ESI	1	6	40	24399	6.48
9	Septin-2	SEPT2_HUMAN	Q15019	MALDI	15	49	145	41487	6.15
10	Alpha-enolase	ENOA_HUMAN	P06733	MALDI	22	56	154	47169	7.01
11	Gamma-enolase	ENOG_HUMAN	P09104	MALDI	15	49	177	47269	4.91
12	14-3-3 protein gamma	1433G_HUMAN	P61981	ESI	4	17	36	28303	4.80
13	14-3-3 protein epsilon	1433E_HUMAN	P62258	ESI	16	45	345	29174	4.63
14	Protein RUFY3 (single axon-regulated protein)	RUFY3_HUMAN	Q7L099	ESI	11	22	57	52965	5.36
15	Dihydropyrimidinase-related protein 2 (CRMP2)	DPYL2_HUMAN	Q16555	MALDI	13	25	132	58163	5.77

### Western immunoblot analyses of 2D-DIGE identified proteins with neurodegeneration relevance

#### Oxidative stress related proteins

**Figure 3:**
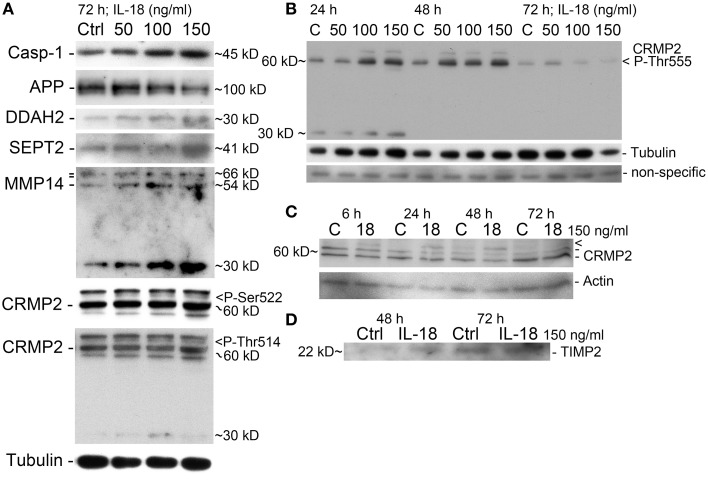
**Western immunoblot verification of the selected targets. (A)** IL-18 induced our apoptosis marker caspase-1 in a concentration dependent manner, with APP level reduced in 72 h treated cells. DDAH2 and SEPT2 levels are increased as well as processed MMP14 (54 kD) and precursor MMP14 (above, 66 kD, propeptide 64 kD). The 30 kD form may be some a non-typical cleavage form of MMP14 (antibody detects aa 511–582 at C-terminus). CRMP2 P-Ser522 specific antibody detected a faint concentration dependent band (>). CRMP2 P-Thr514 also increase (>), with the intensity of CRMP2 different to that of CRMP-2 P-Ser522. Also an additional band about 30 kD was detectable. All the results are from the same membrane. **(B)** CRMP2 also showed increased phosphorylation at Thr555 at 24 and 48 h time-points, after which it reduced, as detected by specific antibody. The band about 30 kD, was evident only at 24 h. Tubulin shows some co-regulation. Non-specific band indicates evenness of sample loading. **(C)** CRMP2 was studied also with non-phosphor specific antibody after 150 ng/ml IL-18 treatment. CRMP2 (isoform 1: 62 kD, isoform 2: 58 kD) were unaffected, but high MW-form above was IL-18 responsive (>), which may be a modified form of CRMP2. CRMP is phosphorylatable at several sites but other modifications may also exist. Actin was used as a loading control. **(D)** IL-18 at 150 ng/ml also increased TIMP2 levels in the culture medium of SH-SY5Y cells vs. untreated control.

Due to the modest changes in expression level observed in 2D-DIGE, the results were verified by WIB with an increased number of repeats along with an additional 6 h time-point. Of the identified proteins, our interest focused on those involved in the oxidative damage evident in AD brains. The WIB data have been collated in Table 4. Protein levels of cytoplasmic peroxiredoxin (PRX) 2 were reduced by 24.8% at the 48 h time-point, with mainly mitochondrial PRX3 showing a pronounced 542% increase at 6 h in IL-18 treated cells, compared to untreated controls. Cytoplasmic vesicle-localized PRX6 (Acidic calcium-independent phospholipase A2) was also increased by 124% at 6 h upon IL-18 treatment. The atypical peroxiredoxin-like peroxidase DJ-1 (Park-7), which also prevents synuclein aggregation (Baulac et al., 2009), was reduced by 24% in 48 h treated cells. The levels of another type of antioxidant, cytoplasmic biliverdin reductase A (BLVRA), showed a 91% increase at the 6 h treatment time-point. DDAH2, involved in the nitric oxide (NO) pathway as well as in apoptosis regulation (Wang et al., 2009), was increased by 40% at 48 h in IL-18 treated cells vs. untreated controls. IL-18 showed a concentration dependent DDAH2 increase at 72 h (Figure 3A). DDAH1 also showed an increase at the 72 h timepoint in the 2D-DIGE analyses, but its expression was not further analyzed by WIB.

**Table 4:** **Protein expression level changes of the selected targets in differentiated SH-SY5Y cells**.

	**6 h**				**24 h**				**48 h**				**72 h**			
	**Mean % ± s.e.m**.	***n***	***p*, T-t**	***p*, M-W**	**Mean ± s.e.m**.	***n***	***p*, T-t**	***p*, M-W**	**Mean ± s.e.m**.	***n***	***p*, T-t**	***p*, M-W**	**Mean ± s.e.m**.	***n***	***p*, T-t**	***p*, M-W**
PRX2					−1.1 ± 17.4	5	0.952	0.095	−24.8 ± 8.6	5	**0.044**	0.095	3.0 ± 24.3	5	0.909	0.577
PRX3	542.3 ± 72.8	6	**0.001**	**0.002**	43.8 ± 21.8	15	0.074	0.096	90.4 ± 61.4	15	0.179	0.319	12.2 ± 14.5	15	0.431	0.319
PRX6	123.8 ± 37.5	11	**0.008**	**0.001**	2.3 ± 10.3	16	0.825	0.519	2.7 ± 12.0	16	0.823	0.519	10.6 ± 14.6	17	0.481	0.348
DJ-1	13.9 ± 14.6	5	0.396	1.000	2.5 ± 14.3	6	0.868	0.305	−23.9 ± 7.5	6	**0.025**	**0.040**	9.2 ± 22.0	6	0.695	0.305
BLVRA	90.8 ± 29.7	11	**0.012**	**0.007**	38.8 ± 21.8	17	0.096	0.348	−7.6 ± 9.7	17	0.445	0.118	33.2 ± 34.3	17	0.347	0.754
DDAH2	75.8 ± 60.9	6	0.269	1.000	37.5 ± 29.5	11	0.116	0.247	40.3 ± 18.9	11	**0.016**	**0.007**	9.5 ± 22.6	11	0.582	0.247
TIMP2	216.9 ± 109.7	5	0.119	0.095	76.6 ± 20.2	10	**0.004**	**0.001**	70.3 ± 21.7	10	**0.010**	**0.001**	37.3 ± 29.7	10	0.240	1.000
MMP14	29.4 ± 11.3	6	**0.048**	**0.002**	28.8 ± 19.2	10	0.169	1.000	15.4 ± 17.4	11	0.396	0.247	89.3 ± 39.3	11	**0.046**	**0.007**
SEPT2	81.3 ± 48.6	4	0.392	0.219	−28.2 ± 16.6	9	**0.039**	**0.003**	10.6 ± 19.3	10	0.913	0.419	90.0 ± 40.9	9	0.097	**0.034**
ENOA	32.4 ± 40.5	6	0.459	1.000	−5.4 ± 5.1	12	0.313	0.459	13.7 ± 10.2	12	0.204	0.459	−26.2 ± 9.1	12	**0.015**	**<0.000**
ENOG					46.4 ± 14.7	5	**0.034**	0.095	73.3 ± 54.6	5	0.251	0.095	9.2 ± 23.0	5	0.711	0.577
14-3-3γ	111.8 ± 50.2	6	0.076	**0.040**	22.5 ± 17.6	8	0.241	0.369	29.9 ± 14.1	8	0.071	0.073	11.3 ± 16.8	8	0.521	1.000
14-3-3ε	−16.1 ± 15.5	6	0.347	0.305	42.5 ± 12.1	10	**0.006**	**0.012**	21.5 ± 20.2	11	0.312	0.700	−7.1 ± 10.1	11	0.498	0.700

The results are expressed as a Mean %.

Mean %: IL-18 treated/untreated control x100-100.

s.e.m., standard error of mean; n, number of separate repeats.

p, p-value [T-test (paired), T-t; Mann–Whitney U-test, M-W]; significant p-values marked as bold (0.05, 0.01, 0.001).

**Figure 4:**
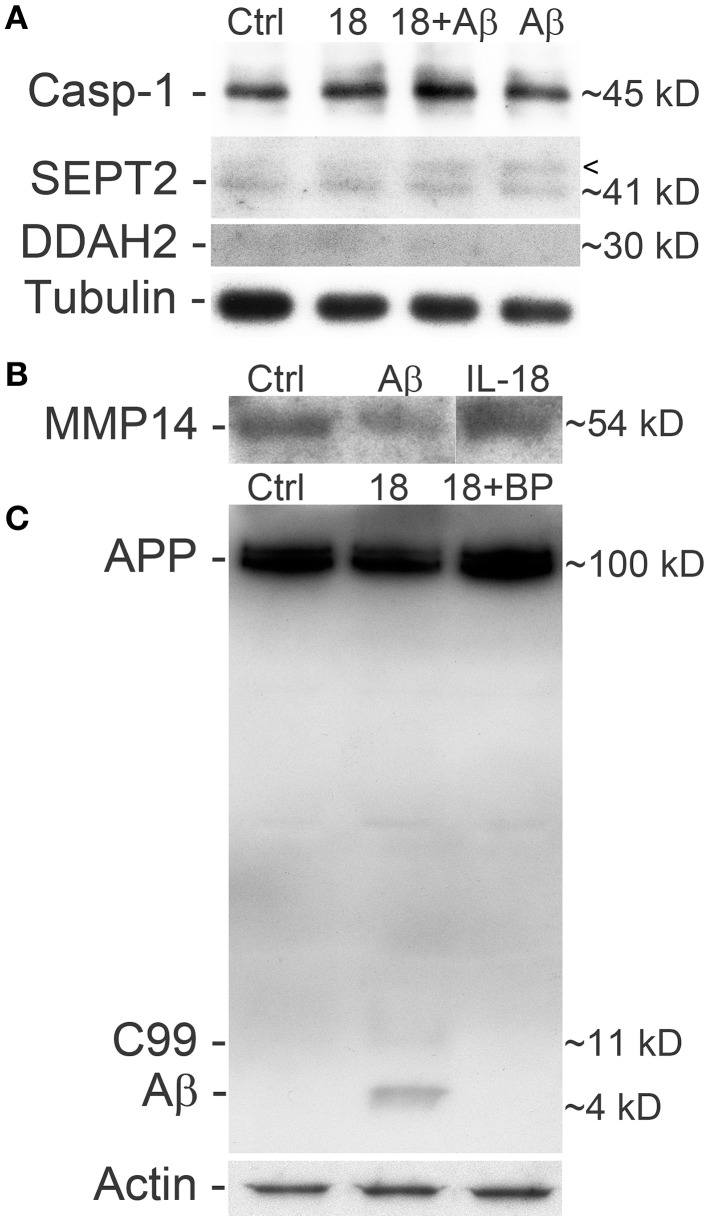
**Differentiated SH-SY5Y cells treated with IL-18 and/or Aβ42 and blocked with IL-18BP. (A)** Treatment with IL-18 (100 ng/ml) or 1 μM Aβ42 increased caspase-1 expression, especially synergistically at 72 h. SEPT2 analysis showed an extra band above the 41 kD main band, which is particularly strong in Aβ42 treated cells and IL-18 + Aβ42 treated cells, compared to untreated control. Although the results of DDAH2 are faint, they indicate increased DDAH2 in IL-18 treated cells whereas treatment with IL-18 + Aβ42, and particularly Aβ42, seemed to reduce its levels vs. control. Tubulin was a loading control. The results are from the same membrane. The appearance of these cells is shown in our previous paper (Sutinen et al., 2012). **(B)** 1 μM Aβ42 decreased soluble MMP14 levels in the SH-SY5Y culture medium. **(C)** IL-18 at 100 ng/ml treatment for 72 h also increased APP processing to C99 and Aβ, which was blocked by 0.5 μg/ml IL-18BP. Actin was used as a loading control.

A concentration of 150 ng/ml of IL-18 was used in these experiments given our previous work showing that IL-18 does not seem to trigger apoptosis at a concentration of 100 ng/ml (Ojala et al., 2008). This may be due to increased antioxidative protective enzymes as well as the anti-apoptotic Bcl-xL, which we have shown previously to be increased by IL-18 (Sutinen et al., 2012). Also the presence of BDNF during these experiments will have protective effects, given our preliminary test showing that the removal of BDNF led to cells detaching from the culture wells as well as increasing vulnerability to cell death. Here we used caspase-1 as a marker for apoptosis initiation showing that IL-18 increased caspase-1 in a concentration dependent manner (Figure 3A). Increased caspase-1 expression was also detectable in samples from our previous study (Sutinen et al., 2012), where the cells were treated with 1 μM Aβ42 with or without 100 ng/ml IL-18 or with IL-18 alone (Figure 4A). IL-18 as 100 ng/ml for 72 h increased caspase-1 protein expression by 34.9% [s.e.m. ±10.9; *n* = 5; *p* = 0.005 (M-W)] and together with 1 μM Aβ42 by 44.5% (±22.2; *n* = 5; *p* = 0.095), whereas 1 μM Aβ42 as such by 23.3% (±22.9; *n* = 5; *p* = 0.095).

#### Amyloid-β related proteins

We found changes in Aβ metabolism and its aggregation affecting targets. APP levels showed an IL-18 concentration depended reduction in 72 h treated cells (Figure 3A). IL-18 as 150 ng/ml for 72 h reduced protein levels of APP [mean -47.5%; s.e.m. ±9.7; *n* = 5; *p* = 0.005 (M-W)]. Further, as shown previously in a separate experiment (Sutinen et al., 2012), IL-18 treatment induced expression of Aβ and this effect was blocked by IL-18BP in 72 h treated cells (Figure 4C). Also Septin-2 (SEPT2), capable of interacting with Aβ aggregates (Pissuti Damalio et al., 2012), decreased by 28% at 24 h following IL-18 treatment but increased to 90.0% after 72 h of treatment. The increase at 72 h was concentration dependent (Figure 3A). Aβ42 at 1 μM (4.5 kD) with or without 100 ng/ml IL-18 increased the higher MW form of SEPT2 (approximately 46 kD), compared to untreated control (41 kD) (Figure 4A), suggesting interactions of Aβ with SEPT2.

Increased tissue inhibitor of metalloproteinase-2 (TIMP2) levels are evident in AD and, through the inhibition of MMPs, can regulate Aβ degradation (Liao and Van Nostrand, 2010; Merlo and Sortino, 2012). In our studies TIMP2 increased by 77% at the 24 h time-point and by 70% at 48 h in IL-18 treated cells. A slight increase in TIMP2 was also detectable in the culture medium after 48 or 72 h following IL-18 treatment (Figure 3D). Although not detected in 2D-DIGE we examined membrane type 1 metalloproteinase (MT1-MMP/MMP-14), which is a target for TIMP2 and can activate MMP2, possibly important in Aβ degradation (Merlo and Sortino, 2012). Protein levels of MMP14 increased by 29% following 6 h of IL-18 and by 89% in the 72 h treated cells, showing an IL-18 concentration dependent effect (Figure 3A). However, whereas IL-18 slightly increased the soluble form of MMP14 in the culture medium, 1 μM Aβ42 treatment for 72 h reduced MMP14 levels (Figure 4B).

#### Other neurodegenerative diseases related proteins

The other interesting targets identified included enolase and 14-3-3 proteins, known to be altered in AD (Fountoulakis et al., 1999; Butterfield and Bader Lange, 2009). Carbon-oxygen lyase α-enolase (ENOA), involved in glucose metabolism, declined by 26% over 72 h of IL-18 treatment of the cells whereas γ-enolase (ENOG), a neurotrophic-like factor promoting growth, differentiation, survival and regeneration of neurons (Hafner et al., 2012), increased by 46% following 24 h of treatment, compared to controls. 14-3-3ε, involved in brain development and neuronal migration (Toyo-oka et al., 2003), increased by 43% in 24 h IL-18 treated cells, whereas the 14-3-3γ isoform, which displays similar functions, increased by 112%, but only at 6 h of IL-18 treatment.

Increased expression of RUFY3 (single axon-regulated protein) (Mori et al., 2007) was also detected after 72 h in IL-18 treated cells under 2D-DIGE, but this finding was not verified by WIB. Collapsin response mediator protein 2 (CRMP2; Dihydropyrimidinase-related protein 2) was examined given its variety of roles in neurons including development, polarity and neuron projection morphogenesis, axon growth and guidance, neuronal growth cone collapse, and cell migration (Higurashi et al., 2012). We found CRMP2 (62.3 kD, isoform 1; 58.2 kD isoform 2) to be modified at least at its Ser522, Thr514, and Thr555 sites (Figures 3A,B). Also, a higher MW-form of CRMP2, induced by 150 ng/ml IL-18 treatment, was detectable (Figure 3C). CRMP2 is modified by phosphorylation of several sites, and possibly also in other ways.

#### Protein levels of DDAH2, SEPT2, and MMP14 were altered in the AD brain

**Figure 5:**
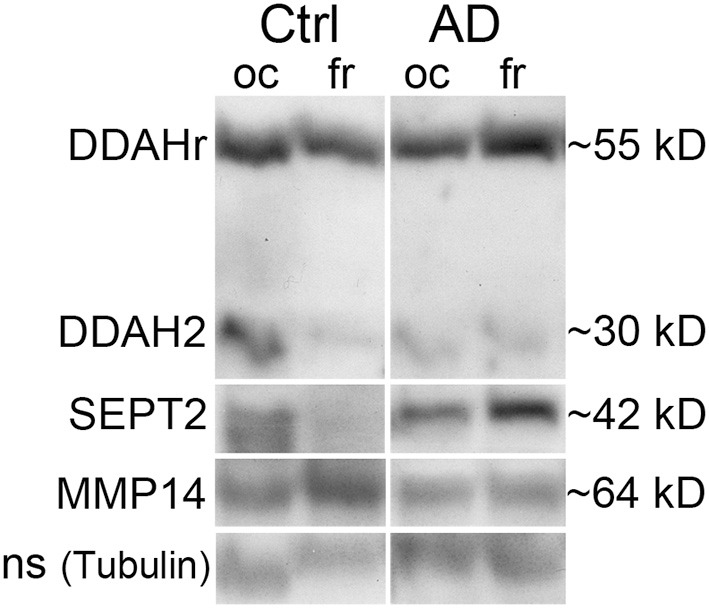
**Western immunoblot example set of AD and control brain post-mortem samples**. DDAH, SEPT2, and MMP14 were examined from the age matched samples. The shown sample sets are from one control and one AD subjects, the shown bands being detected from the same membrane after stripping. DDAH2 reduced in oc lobe of AD patients but increased in fr lobe, similarly as the around 40 kD form. The around 55 kD form is possibly complexed DDAH2 (29 kD) or DDAH1 (the size of a biological form of DDAH1 is 37 kD), as shown in 2D-gel (Figure 1, spot 6). SEPT2 increased and MMP14 reduced in fr lobe in AD. With tubulin antibody detected non-specific (ns) band was a loading control due to excess intensity of tubulin. Ctrl, control; AD, Alzheimer's disease; oc, occipital lobe sample, fr, frontal lobe sample.

Based on the above, the most interesting targets were examined from frontal and occipital lobe post-mortem samples of AD patients and healthy age matched controls. An example set of DDAH, SEPT2 and MMP14 is shown in Figure 5, and it describes general changes in occipital and frontal lobe samples from one control and one AD patient, examined from the same WIB membrane. As collected in Table 5, we found that in occipital lobe, DDAH2 (approximately 29 kD) and SEPT2 levels reduced about 77% in AD patients, whereas DDAH2 antibody reactive protein (DDAHr; approximately 55 kD) increased in frontal lobe almost 140% compared to healthy controls. In healthy controls, DDAHr (approximately 55 kD) was about 50% less in frontal lobe than in occipital lobe. This 55 kD DDAHr form requires further studies. In AD patients, MMP14 was increased around 80% compared to healthy controls, although its general level in AD patients seemed to be less than in healthy controls in both lobes. Therefore, the general slight reduction of MMP14 levels in AD may alter for instance the Aβ degradation pathways. The specific role of MMP14 in AD pathogenesis requires further investigation. Due to the biological variations of human samples, more definite results of the given targets require higher number of AD and control samples.

**Table 5:** **Expression level changes of the selected target proteins in occipital and frontal lobes of AD patients and healthy controls**.

	**Mean ± s.e.m**.	***n***	**Mean ± s.e.m**.	***n***	**Mean %**	***p*, T-t**	***p*, M-W**	**Mean %**	***p*, T-t**	***p*, M-W**
	**Ctrl, oc**		**AD, oc**		**AD/Ctrl, oc**			**Ctrl, fr/oc**		
DDAHr ~55 kD	1.60 ± 0.40	6	1.39 ± 0.28	9	−12.7	0.673	0.749	−49.0	**0.049**	0.109
DDAH2 ~29 kD	5.44 ± 2.23	4	1.22 ± 0.65	5	−77.6	0.084	**0.050**	−78.9	0.055	0.083
SEPT2	4.57 ± 1.92	4	1.03 ± 0.43	5	−77.5	0.084	**0.027**	−56.3	0.145	0.248
MMP14	1.54 ± 0.62	6	0.94 ± 0.12	9	−38.8	0.272	0.631	48.8	0.168	0.200
	**Ctrl, fr**		**AD, fr**		**AD/Ctrl, fr**			**AD, fr/oc**		
DDAHr ~55 kD	0.82 ± 0.15	6	1.95 ± 0.32	9	138.9	**0.018**	**0.010**	39.6	0.107	0.145
DDAH2 ~29 kD	1.15 ± 0.52	4	1.01 ± 0.40	5	−11.9	0.837	1.000	−17.3	0.394	0.917
SEPT2	1.99 ± 1.10	4	1.26 ± 0.50	5	−37.1	0.531	0.806	21.8	0.371	0.754
MMP14	2.29 ± 0.40	6	1.71 ± 0.36	9	−25.3	0.309	0.262	81.6	**0.029**	**0.019**

Mean: calculated from the numerical band densities [Graph density × area (G/DxA), expressed as a percentage of the total area scanned] after normalization with tubulin; numerical value describes the relative intensity of the examined band.

Mean %: AD/Ctrl or frontal/occipital x100-100.

s.e.m., standard error of mean; n, number of individuals.

P, p-value [T-test, T-t (paired); Mann–Whitney U-test, M-W]; significant p-values marked as bold (0.05, 0.01, 0.001).

Ctrl, healthy control; AD, Alzheimer's disease; oc, occipital lobe; fr, frontal lobe.

In conclusion, the results of many neuronal cell types including post mortem AD samples do not correlate well in our living one cell type neuronal model in this number of repeats. However, our model may describe the events in neurons *in vivo* when acute high levels or even chronic lower levels of IL-18 are present. Our earlier concentration studies have suggested that lower doses of IL-18 (50 ng/ml) also have similar effects than higher 100 or 150 ng/ml doses, but in a longer timeframe, apparent also in some level in Figures 3A,B. For the maintenance of the cells, longer than 72 h incubation time would require the culture medium change leading to distortions of the experiment as well as the results.

## Discussion

**Figure 6:**
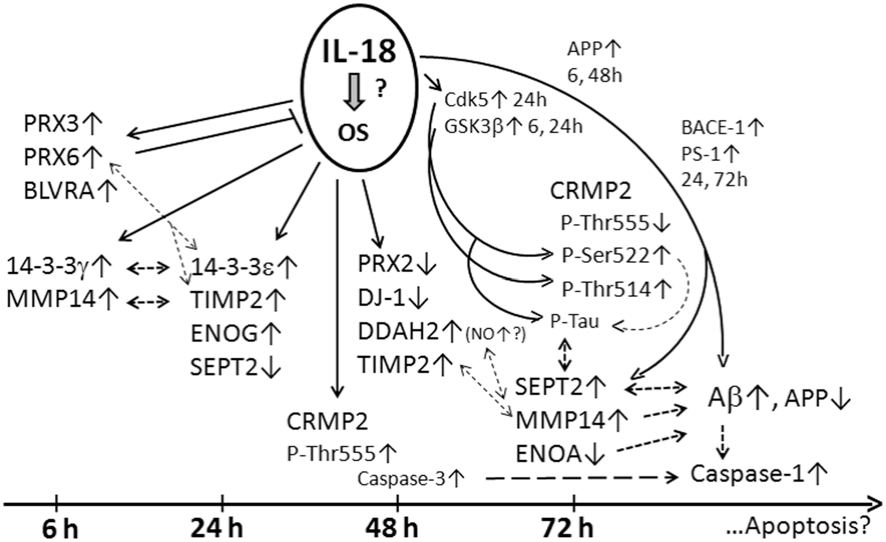
**Summary figure of the WIB results**. IL-18 may induce OS due to increase of antioxidative **PRX3**, -6, and **BLVRA** in SH-SY5Y cells. **14-3-3**γ (modulator of apoptosis; intracellular trafficking; cell cycle control; signal transduction; and metabolism), **14-3-3**ε (anti-apoptotic; a component of the prion/amyloid deposits of Gerstmann-Straüssler-Scheinker disease). 14-3-3ε can bind PRX6, translocating PRX6 to lysosomes when MAPK is activated. Both 14-3-3γ and -ε can prevent α-synuclein inclusion formation. 14-3-3 proteins can complex with tau and GSK3β promoting tau phosphorylation and NFT formation. 14-3-3ε can increase MMP2. **TIMP2** (MMP inactivator), **MMP14** (a physiological activator for pro-MMP2 complexed with TIMP2). IL-18 inducible MMP2 and MMP9 are also expressed in SH-SY5Y cells. MMP2, -9 and soluble MMP14 can participate in Aβ degradation. Increased PRX6 is linked with increased MMP9 and uPA, and decreased TIMP2 expression. uPA and tPA are expressed in SH-SY5Y cells, and both, via plasminogen, increase protease plasmin, also able degrading Aβ and regulating MMP levels. Active plasmin can remodel directly and indirectly, via activation of MMPs, the ECM. **ENOG** (glycolysis; a neurotrophic-like factor promoting growth, differentiation, survival, and regeneration of neurons). The neurotrophic activity is mediated by the C-terminus which activates PI3K/Akt and MAPK/ERK. Cathepsin X, which activity increases during aging and interacts also with Aβ-plaques, cleaves the C-terminus of ENOG and ENOA, impairing the survival and neuritogenesis of neuronal cells. **DJ-1** (scavenges H_2_O_2_; modulates Cu/Zn-SOD; a redox-sensitive chaperone; prevents α-SNC aggregation; detectable in tau inclusions; loss of function leads to neurodegeneration, likely mediated by mitochondria). DDAH (regulates apoptosis; controls cellular methylarginine concentration by hydrolyzing NOS inhibitors ADMA and MMA). **DDAH** can increase NOS activity, NO production and resultant increase in nitrosative stress. NO also regulates learning and memory. ONOO- can activate MMPs. **ENOA** (glycolysis; plasminogen receptor). Interaction of plasminogen with ENOA enhances its activation to plasmin by tPA or uPA. Modifications of ENOA can lead to its catalytic inactivation, which can alter its role as a plasminogen receptor, effecting ECM remodeling, neuronal survival and Aβ degradation. **SEPT2** (interacts with Aβ; linked to pathological features of Aβ plaques and NFTs; filament-forming cytoskeletal GTPase that can modulate cytokinesis, cell division, exocytosis, and membrane trafficking). Generally, Septins form hetero-oligomeric complexes which assemble into filaments, mediated by the GTPase activity. **CRMP2** (mediates Sem3A signaling in the nervous system; interacts e.g., with tubulin, Numb, kinesin1, and Sra1; promotes axon elongation; phosphorylated by several Ser/Thr kinases (ROCK: Thr555, Cdk5: Ser522, GSK3β: Ser518, Thr514,509). Ser522-phosphorylation is essential for sequential phophorylations by GSK3β at Ser518 and Thr514,509. These phosphorylation steps are required in Sem3A signaling. The phosphorylations disrupt the interaction of CRMP2 with tubulin or Numb. CRMP2 hyperphosphorylation is an early event in AD progression and occurs downstream of APP, but neither excessive Aβ42 peptide nor neurotoxicity alone are sufficient to promote it. CRMP2 hyperphosphorylated at Thr509 and Ser518,522 is also present in NFTs. IL-18 can induce **Cdk5** and **GSK3**β and **caspase-3** (Ojala et al., 2008) as well as **APP**, **BACE-1**, and **PS-1** (Sutinen et al., 2012) marked as smaller font. Interactions are shown with dashed lines.

In the present study, we found that IL-18 had time-dependent effects on protein expression profiles in differentiated neuron-like SH-SY5Y cells. As summarized in Figure 6, our study shows that IL-18 either by itself or through induction of OS, differentially induces protective enzymes, as well as proteins associated with Aβ degradation vs. accumulation and apoptosis. IL-18 was shown to regulate targets affecting glucose metabolism, survival and regeneration, neuronal differentiation, and brain development. Many of these proteins have previously been studied in different AD brain regions, and are linked to AD/dementia (Korolainen et al., 2010). The findings are also in line with our earlier studies showing increased IL-18 in the brain of AD patients (Ojala et al., 2009), in turn contributing to many time-dependent protein changes, which can contribute to the pathogenesis of AD, including via NFT and Aβ-plaque formation (Ojala et al., 2008; Sutinen et al., 2012).

OS plays an important role in the pathogenesis of numerous age-related neurodegenerative diseases including multiple sclerosis (MScl) and Parkinson's disease (PD) (Anderson and Maes, 2014), as well as AD, by inducing cell damage and wider inflammatory processes (Krapfenbauer et al., 2003). Uncontrolled OS can lead to apoptosis but it can also cause sublethal damages that induce regenerative mechanisms (Ojala et al., 2008), leading to protein alterations typical of AD. However, Aβ itself can also induce OS (Opazo et al., 2002) and, as we showed, IL-18 can induce Aβ-production. Oxidatively modified proteins in turn are associated with tau and Aβ pathology (von Bernhardi and Eugenín, 2012). The brain is highly sensitive to OS due to its richness of fatty acids that can undergo peroxidation and its high oxygen demand, coupled to a relative scarcity of robust antioxidant systems (Lee et al., 2010). Among the multiple mechanisms and triggers by which OS can accumulate, inflammatory cytokines can sustain oxidative and nitrosative stress, leading eventually to increased levels of oxidation-altered metabolites. As such, understanding of the consequences of inflammation and OS is important since it may elucidate the initial events underlying AD.

We found expression changes in PRXs after the IL-18 treatments. PRXs (PRX1-6) are a family of multifunctional antioxidant thioredoxin-dependent peroxidases that are differentially localized intracellularly, with further variation across different cell types (Hanschmann et al., 2013). The major functions of PRXs involve cellular protection against OS, including ROS-mediated DNA-fragmentation, modulation of intracellular signaling cascades that utilize hydrogen peroxide as a second messenger molecule and the regulation of cell proliferation. PRX2 is expressed exclusively in neurons (Krapfenbauer et al., 2003). PRX3 levels are decreased in the AD brain, and since mitochondria are generally also extensively damaged in AD, leakage may contribute to its reduction (Krapfenbauer et al., 2003). PRX6 is increased in astrocytes, diffuse plaques and in neuritic plaques, but not in AD neurons (Power et al., 2008). It can reduce hydrogen peroxide as well as short chain organic, fatty acids and phospholipid hydroperoxides, indicating its importance in the regulation of phospholipid turnover (Fisher, 2011). PRX2 deficient neurons are particularly sensitive to hydrogen peroxide induced cell death. PRX2, like anti-apoptotic Bcl-2 and Bcl-xL proteins, can also interact with presenilin-1 (PS-1), thereby being involved in processing of APP to Aβ. This binding can provide protection against neuronal apoptosis (Alberici et al., 1999; Zhou et al., 2002).

DJ-1 reduced by IL-18 treatment. DJ-1, a susceptibility gene in PD, can scavenge hydrogen peroxide and function as a redox-sensitive chaperone (Batelli et al., 2008; Baulac et al., 2009). It can also regulate oxidant status via the modulation of Cu/Zn-superoxide dismutase-1 (Cu/Zn-SOD) levels (Wang et al., 2011), as well as preventing α-synuclein aggregation (Batelli et al., 2008). DJ-1 is also detectable in tau inclusions (Rizzu et al., 2004) and further, it is required to correct mitochondrial morphology and function, as well as the autophagy of dysfunctional mitochondria. Therefore, loss of its function generally leads to neurodegeneration, likely mediated by mitochondria (Irrcher et al., 2010). As a summary, the decrease in DJ-1, induced by IL-18, is likely to have wide ranging effects, including mitochondrial function and antioxidant regulation.

IL-18 treatment increased BLVRA levels, as evident also in AD (Barone et al., 2011). Evolutionarily conserved BLVR and heme oxygenase (HO) have an essential role in the fight against OS. BLVR is expressed in all tissues as BLVRA (dominant in adults) and BLVRB (dominant in fetal state) (Kim and Park, 2012). Multifunctional BLVR is regulated by a number of factors including biliverdin and phosphorylation, which in turn are regulated by OS and free radicals (Wegiel and Otterbein, 2012). HO is needed to cleave the heme ring to form biliverdin (BV), with soluble BLVR converting BV to the potent antioxidant bilirubin (BR) (Jansen et al., 2010). Both BR and BV are potent scavengers of peroxyl radicals (Jansen and Daiber, 2012). BR can also interact with and neutralize NO radicals leading to the formation of NO-bilirubin (Barone et al., 2009), whereas BV, through NO-dependent nuclear translocation of BLVR, inhibits Toll-like receptor-4 (TLR4) expression (Wegiel et al., 2011), another factor known to regulate AD susceptibility (Chen et al., 2012). Interestingly, due to the lipophilic nature of BR, it has suggested to be particularly effective against lipophilic ROS (Jansen and Daiber, 2012). BLVR is also known to function as a Ser/Thr/Tyr-kinase and transcription factor as well as in cell-signaling, mediated by protein kinase C (PKC), mitogen activated protein kinase (MAPK), and phophatidylinositol 3-kinase (PI3K) (Lerner-Marmarosh et al., 2005; Gibbs et al., 2012; Kim and Park, 2012). Therefore, the increase in BLVR by IL-18 is likely to modulate neuronal antioxidants, transcription and intracellular signaling pathways.

Protein levels of DDAH were increased in IL-18 treated cells as well as the higher MW DDAHr within the frontal lobes of AD patients. DDAH controls cellular methylarginine concentration by hydrolyzing N(G),N(G)-dimethyl-L-arginine (ADMA), and N(G)-monomethyl-L-arginine (MMA) which act as inhibitors for nitric oxide synthase (NOS) (Selley, 2004; Pope et al., 2009). Catabolization of these endogenous inhibitors by DDAH may increase NOS activity and NO production with resultant increases in nitrosative stress. NO also has an important role in synaptic events regulating learning and memory, and its dysregulation may lead to synaptic dysfunction (Selley, 2004). Further, NO is a vasodilator and can regulate blood pressure (Dayoub et al., 2008). DDAH1 localizes uniformly in the cytosol and nucleus, whereas DDAH2 is in the cytosol (Birdsey et al., 2000), but may also translocate to mitochondria upon IL-1β stimulation (Cillero-Pastor et al., 2012). Interestingly, ADMA/DDAH pathway can regulate angiogenesis (Fiedler et al., 2009). Overall, the increase observed in DDAH1 detected in 2D-gels and DDAH2 (29 kD) following IL-18 treatment will alter NOS, NO, and nitrosative stress, as well as NO related signaling processes. DDAHr (around 55 kD) requires further studies.

TIMP2 and MMP14 levels were time-dependently increased in IL-18 treated cells. TIMP2 inactivates matrix MMPs by covering their catalytic zinc cofactor site (Brew and Nagase, 2010). On the other hand, MMP14 is a physiological activator for several MMPs, including for pro-MMP2, complexed with TIMP2 (Lehti et al., 1998; Itoh et al., 2001). MMPs can also be activated by the free radical peroxynitrite (ONOO-) (Ridnour et al., 2012). IL-18 inducible MMP2 and MMP9 are expressed in SH-SY5Y cells (Ishida et al., 2004; Chandrasekar et al., 2006; Merlo and Sortino, 2012), and further, MMP2, -9 as well as a soluble MMP14 form can participate in the degradation of Aβ (Liao and Van Nostrand, 2010; Merlo and Sortino, 2012). However, increased PRX6 expression is associated with increased levels of MMP9 and the urokinase-type plasminogen activator (uPA) as well as decreased TIMP2 (Chang et al., 2007; Lee et al., 2009b). Both uPA and tissue-type plasminogen activator (tPA) are also expressed in SH-SY5Y cells (Neuman et al., 1989), and both of them, via plasminogen, increase the protease plasmin, also able to degrade Aβ (Tucker et al., 2000) and regulate MMP levels. Active plasmin can also remodel the extracellular matrix (ECM) directly, as well as indirectly via the activation of MMPs (Baramova et al., 1997). In AD patients, MMP14 levels are higher in the frontal lobe compared to the occipital lobe, but seemed to be less than in healthy controls. As such, IL-18 regulation of PRX6, TIMP2, NO, MMPs and MMP14 may be associated with the regulation of Aβ production as well as plasmin and MMP mediated Aβ degradation.

Whereas ENOG levels increased, ENOA levels declined time-dependently in IL-18 treated cells. Homo- or heterodimeric ENOs are glycolytic enzymes, which are also associated with hypoxia and ischemia as well as AD (Sultana et al., 2007; Butterfield and Bader Lange, 2009). ENOA is consistently found to be up-regulated in mild cognitive impairment and AD, in which glucose hypometabolism and upregulation of glycolytic enzymes are predominant features (Castegna et al., 2002). ENOA can be glycosylated (Owen et al., 2009), oxidized (Castegna et al., 2002), glutathionylated (Newman et al., 2007), or nitrated (Reed et al., 2009), which, when elevated, lead to its catalytic inactivation. Depending on the pathophysiological condition of the cells, ENOA can also function as a plasminogen receptor on the surface of several cell types (Díaz-Ramos et al., 2012). Interaction of plasminogen with ENOA enhances its activation to plasmin by tPA or uPA (Sinniger et al., 1999). However, ENOA modifications that lead to its catalytic inactivation are also likely to alter its role as a plasminogen receptor (Gentile et al., 2009), with consequences for ECM remodeling, neuronal survival and Aβ degradation. Although ENOG levels are increased markedly, for instance in cardiovascular disease, cerebral trauma, brain tumors, and Creutzfeldt-Jakob disease, it can also function as a neurotrophic-like factor promoting growth, differentiation, survival, and regeneration of neurons after translocation and binding to the plasma membrane (Aksamit et al., 2001; Hafner et al., 2010, 2012). Its neurotrophic activity is regulated by the C-terminal peptide which activates the PI3K/Akt and MAPK/ERK signaling pathways (Hafner et al., 2012). Cathepsin X, the activation of which increases in an age-dependent manner and is also associated with Aβ-plaques (Wendt et al., 2007), cleaves the C-terminal amino acids of ENOG as well as ENOA, impairing the survival and neuritogenesis of neuronal cells (Obermajer et al., 2009). Whether cathepsin X is inducible by IL-18 is unknown.

We found time-dependent changes in the expression of 14-3-3 proteins. Generally, the 14-3-3 family of proteins is a highly conserved group of molecules that play important roles in apoptosis, intracellular trafficking, cell cycle control, signal transduction, and metabolism (Steinacker et al., 2011). 14-3-3ε can protect against ischemic cerebral infarction and neuronal apoptosis (Wu et al., 2009), but it is also a component of the prion protein amyloid deposits of Gerstmann-Straüssler-Scheinker disease (Di Fede et al., 2007) indicating a role in amyloid regulation in other neurological conditions. 14-3-3ε can also bind PRX6, which leads to translocation of PRX6 to lysosomal organelles in the presence of MAPK activity (Sorokina et al., 2011). 14-3-3γ and 14-3-3ε also have binary interactions with leucine-rich repeat Ser/Thr-protein kinase 2 (LRRK2), detected in α-synuclein positive Lewy bodies, as well as in Hirano bodies in AD (Perry et al., 2008; Li et al., 2011). Both 14-3-3γ and -ε can prevent α-synuclein inclusion formation (Yacoubian et al., 2010) and via its association with phosphorylated Bad prevent its apoptotic actions (Koh, 2008). 14-3-3 proteins can also bind simultaneously to tau and GSK3β and promote tau phosphorylation and formation of NFTs (Yuan et al., 2004). Further, 14-3-3ε can increase MMP2 expression via p38 MAPK signaling (Lee et al., 2009a). As such, the different 14-3-3 isoforms have variable effects that may contribute to AD degenerative processes, as well as having wider beneficial effects, which will be modulated by IL-18.

IL-18 regulated SEPT2 expression in time-dependent manner. There was also an indication that SEPT2 levels are increased in the frontal lobe compared to occipital lobe of AD patients, although this requires further investigation. SEPT2, capable of interacting with Aβ aggregates, has been linked with NFTs and pathological features of Aβ plaques in AD (Kinoshita et al., 1998; Pissuti Damalio et al., 2012). SEPT2 is a member of the septin family, which is a conserved group of filament-forming cytoskeletal GTPases. The members form hetero-oligomeric complexes which assemble into filaments in a GTPase activity required manner. As such the septins have a wide range of physiological effects, including roles in the regulation of cytokinesis, cell division, exocytosis, and membrane trafficking (Peterson and Petty, 2010; Mostowy and Cossart, 2012).

The other interesting targets, found in 2D-DIGE, were RUFY3 and CRMP2. RUFY3 is implicated in single axon formation by developing neurons due to its ability to inhibit PI3K in minor neuronal processes, thereby preventing the formation of additional axons (Mori et al., 2007). We also found time-dependent changes in phosphorylation of CRMP2. CRMP2 is a member of the CRMP family and promotes axon elongation in primary hippocampal neurons or SH-SY5Y cells (Cole et al., 2004). CRMP2 seems to be involved in neurodegenerative mechanisms common to AD and quite possibly also other neuroinflammatory conditions, such as MScl (Petratos et al., 2008, 2012). Generally, CRMPs are the major phosphoproteins in the developing brain, and they mediate Semaphorin3A (Sem3A) signaling in the nervous system. They interact with many factors, including tubulin, Numb, kinesin1, and Sra1 (Uchida et al., 2009). For instance CRMP2 is phosphorylated by several Ser/Thr kinases, such as Rho kinase (ROCK), Cdk5, and GSK3β especially at its C-terminal sites. As we showed previously, IL-18 can induce Cdk5 and GSK3β (Ojala et al., 2008), suggesting a role for IL-18 in the regulation of CRMP2. ROCK phosphorylates CRMP2 at Thr555 and Cdk5 at Ser522, with Ser522-phosphorylation being essential for sequential phophorylations by GSK3β at Ser518, Thr514, and Thr509. This sequential phosphorylation of CRMP2 by Cdk5 and GSK3β is a necessary step in Sem3A signaling (Uchida et al., 2009), with the CRMP2 phosphorylations disrupting the interaction with other factors including tubulin or Numb. Further, Sem3A or ROCK pathways seem to independently regulate growth cone collapse (Arimura et al., 2000), whereas phosphorylation sites and levels of CRMP2 may control axon growth by coordinating the stability and configuration of growth cone microtubules (Hur et al., 2011). However, abnormal CRMP2 hyperphosphorylation is an early event in AD progression (Cole et al., 2007) and occurs downstream of APP, but neither excessive Aβ42 peptide nor neurotoxicity alone are sufficient to promote it (Williamson et al., 2011). CRMP2, hyperphosphorylated at Thr509, Ser518, and Ser522, is also present in NFTs (Gu et al., 2000). With axonal growth cones being dynamic extensions of developing axons that seek appropriate synaptic targets, it is plausible that hyperphosphorylated forms of CRMP2 that are evident in AD may represent a blockade of such growth mechanisms in dystrophic/swollen neurites.

In conclusion, the increase in antioxidative enzymes BLVRA, PRX3 and -6 as well as generally neuroprotective 14-3-3 proteins is likely driven either by IL-18 itself or by induction of OS (Figure 6). DDAH2 increases at a later time-point suggest increased nitrosative stress, and reduced DJ-1 increased mitochondrial stress, which combined to Aβ production may lead to activation of caspase-1 and apoptosis. Increased TIMP2 as well as decreased ENOA and 14-3-3ε may indirectly lead to increased Aβ accumulation, whereas increased SEPT2 may enhance Aβ aggregation. Changes in CRMP2 phosphorylation suggest impaired interactions with tubulin. The most interesting novel targets were examined in AD brain samples, where DDAHr (about 55 kD) was increased in frontal lobes of AD patients and general levels of MMP14 appeared to be decreased with this number of repeats. The role of SEPT2 in AD requires further studies. The SH-SY5Y findings are in line with our earlier studies, in which we have shown that IL-18 can induce several time-dependent protein changes that have a role in the pathogenesis of AD including NFT and Aβ-plaque formation (Ojala et al., 2008; Sutinen et al., 2012). Further, in the present study increased antioxidative enzymes seems to be followed by APP processing to Aβ (Sutinen et al., 2012). IL-18 also increased concentration dependently caspase-1 expression, indicating initiation of the apoptotic pathway. Whether APP processing to Aβ in relation to OS or its regulating enzymes is a cause or a consequence of the activation of apoptotic pathway in neurons requires further studies. However, in aged normal human neurons especially, the poorly functioning defense systems may lead to apoptosis and/or enhanced processing of APP to Aβ. Overall, our work strongly suggests that increased IL-18 plays an important role in AD, particularly in overlapping the biological underpinnings of the diseases that increase AD risk, such as obesity, type-2 diabetes and depression as well as when wider AD risk gene alleles are present.

## Author contributions

Johanna O. Ojala carried out the cell culture, Elina M. Sutinen and Johanna O. Ojala the laboratory work including WIBs, and Elina M. Sutinen, Johanna O. Ojala, Minna A. Korolainen, and Jukka Häyrinen the gel- and mass spectrometry analyses; Johanna O. Ojala, Tuula Pirttilä, Elina M. Sutinen, and Minna A. Korolainen contributed to the design of the study; Irina Alafuzoff provided the human samples; Tuula Pirttilä, Elina M. Sutinen, Minna A. Korolainen, and Hilkka Soininen provided the research support. Johanna O. Ojala and Elina M. Sutinen wrote the manuscript. Steven Petratos, Antero Salminen, and Hilkka Soininen had intellectual input to the manuscript. All authors read and approved the final version of the manuscript.

### Conflict of interest statement

The authors declare that the research was conducted in the absence of any commercial or financial relationships that could be construed as a potential conflict of interest.

## Abbreviations

Aβ: Amyloid-β
ACN: Acetonitrile
AD: Alzheimer's disease
ADMA: N(G),N(G)-dimethyl-L-arginine
APP: Amyloid-β precursor protein
ATRA: all-trans retinoic acid
BACE-1: β-site APP cleaving enzyme 1
BDNF: brain derived neurotrophic factor
BLVR: biliverdin reductase
BR: bilirubin
BV: biliverdin
Cdk5: cyclin dependent kinase-5
CHAPS: 3-[(3-cholamidopropyl)dimethylammonio]-1-propanesulphonate
CRMP2: collapsin response mediator protein 2
Cu/Zn-SOD: Cu/Zn-superoxide dismutase-1
DDAH: N(G), N(G)-dimethylarginine dimethylaminohydrolase
DDAHr: DDAH2 antibody reactive protein
2D-DIGE: two-dimensional fluorescence difference gel electrophoresis
DJ-1: Parkinson disease protein 7
DTT: dithiothreitol
ECM: extracellular matrix
EDTA: ethylenediaminetetraacetic acid
ENOA: enolase α
ENOG: enolase γ
ERK: extracellular signal-regulated kinase
ESI: electrospray ionization mass spectrometry
FA: formic acid
GSK3β: glycogen synthase kinase-3β
HO: heme oxygenase
HPLC: High-performance liquid chromatography
IAA: iodoacetamide
ICE: interleukin-ıβ convertin enzyme
IDO: indoleamine 2,3-dioxygenase
IGIF: interferon-γ inducing factor
IL-18BP: interleukin-18 binding protein
IFNγ: interferon-γ
LC-MS: liquid chromatography-mass spectrometry
LRRK2: leucine-rich repeat serine/threonine-protein kinase 2
MALDI: matrix-assisted laser desorption ionization
MAPK: mitogen activated protein kinase
MMA: N(G)-monomethyl-L-arginine
MS: mass spectrometry
MScl: multiple sclerosis
MMP14/MT1-MMP: membrane-type-1 matrix metalloproteinase
M-W: Mann–Whitney *U*-test
MW: molecular weight
NFT: neurofibrillary tangles
NOS: nitric oxide synthase
OS: oxidative stress
PAGE: polyacrylamide gel electrophoresis
PBS: phosphate buffered saline
PD: Parkinson disease
pI: isoelectric point
PI3K: phophatidylinositol 3-kinase
PKC: protein kinase C
PMF: peptide mass fingerprint
PRX: peroxiredoxin
PS-1: presenilin 1
ROS: reactive oxygen species
RT: room temperature
SDS: sodium dodecyl sulfate
s.e.m.: standard error of mean
Sem3A: semaphorin- 3A
SEPT2: Septin-2
TBS: tris buffered saline
TCEP: tris (2-carboxyethyl) phosphine hydrochloride
TFA: trifluoroacetic acid
TIMP2: tissue inhibitor of metalloproteinases 2
TLR4: toll-like receptor 4
TOF: time of flight
tPA: tissue-type plasminogen activator
T-t: Student's *t*-test
uPA: urokinase-type plasminogen activator
WIB: western immunoblotting.
