# Supplementary material for: Interleukin-18 alters protein expressions of neurodegenerative diseases-linked proteins in human SH-SY5Y neuron-like cells
Source: Front Cell Neurosci. 2014 Aug 7;8:214. doi: 10.3389/fncel.2014.00214 (PMC4124869; doi:10.3389/fncel.2014.00214)
Supplement: Supplementary file 1 [file DataSheet1.ZIP › Supplementary Data 2 & Captions.DOCX]

**Supplementary data 1.** In the study used antibodies (listed in Table 1) were tested in different cell lines, and in some cases also in the post mortem sample from brain frontal lobe (Br fr) using WIB. Equal amount of protein (35 μg) was loaded in the gels except Br fr sample (17.5 μg), due to the problems in excess band intensity. Lipopolysaccharide (L) -stimulation induces OS in THP-1 cells (Rushworth et al., 2005; Ventura et al., 2009). We also induced external OS for differentiated SH-SY5Y cells with H_2_O_2_. **PRX2** is expressed in macrophage type of cells and it is inducible by L (Yang et al., 2007; Bast et al., 2010) as well as **PRX3** (Li et al., 2007; 2009), **PRX6** (Bast et al., 2010) and **DJ-1** (Mitsumoto and Nakagawa, 2001; Haniu et al., 2011). **BLVRA** is expressed in the brain and in liver of which HepG2 cells are an example. It is also developmentally regulated (Maines, 1990; Komuro et al., 1996; Perez et al., 2008; Barone et al., 2011). **DDAH2** is the main form in muscle and it is also expressed in macrophage type cells being responsive to L (Leiper et al., 1999; Chen et al., 2008; Sharma et al., 2010). **TIMP2** is expressed in macrophage type cells and quite highly in epithelial Arpe-19 cells. It is also involved in muscle cell differentiation (Lluri and Jaworski, 2005; Stawowy et al., 2005; Li et al., 2010; Wang et al., 2010). **MMP14** is expressed in macrophage type cells and it is inducible by L. It is also involved in muscle cell differentiation (Stawowy et al., 2005; Wang et al, 2010; Nishioka et al., 2012; Lively and Schlichter, 2013). **SEPT2** is expressed in polarizing epithelial cells of which Arpe-19 cells are an example (Dunn et al., 1996; Spiliotis et al., 2008; Bowen et al., 2011). **CRMP2** is expressed mainly in epithelial cells among nonneuronal cells, of which Arpe-19 cells are an example (Yoneda et al., 2012). **ENOA** is expressed in the liver but also in the brain where it is developmentally regulated, as well as in macrophage type cells being inducible by L (Joseph et al., 1996; Wygrecka et al., 2009). **ENOG** is a neuronal form of ENOs and its expression is developmentally regulated (Schengrund and Marangos, 1980; Joseph et al., 1996). **14-3-3γ** is expressed in neurons, THP-1 cells and quite highly in liver of which HepG2 cells are an example (Matsui et al., 2011; Konakahara et al., 2012; Baumgartner et al., 2013; Taurino, et al., 2014). **14-3-3ε** is developmentally regulated, expressed in neurons and it is expressed in muscle and liver (McConnell et al., 1995; Luk et al., 1998; Umahara, et al. 2004; Taurino, et al., 2014). **Casp1** is expressed in neurons, as well as in monocytes and it is inducible by L (Schumann et al. 1998, Adamczak et al., 2014). S, SH-SY5Y, differentiated as described followed by culture for 24 (control for H), 48 or 72h; H, 0.1 mM H_2_O_2_ treatment for 10 min in DMEM w/o FBS and supplements, followed by 24h culture in DMEM (BioWhittaker/Cambrex), supplemented with 5% FBS (FBS; HyClone/Pierce), 2 mM L-glutamine (Cambrex/Lonza), 100 U/ml penicillin and 10 µg/mL streptomycin (Cambrex/Lonza). Snb, undifferentiated SH-SY5Y neuroblastoma; THP, THP-1 human acute monocytic leukemia cells; L, stimulated with 5 μg/mL lipopolysaccharide (E. coli, 055:B5, Sigma, Aldrich) for 24h; Hep, HepG2 human liver hepatocellular carcinoma cell line; mb, L6 rat myoblasts; mt, L6 partially, for 3 days differentiated myotubes; Arpe, Arpe-19 human retinal pigment epithelia cells (Dunn et al., 1996). Actin is shown as a loading control, although there is a varying amount of actin in different cell types and therefore is usable mainly in comparison of the untreated/treated pairs.

**Supplementary data 2.**


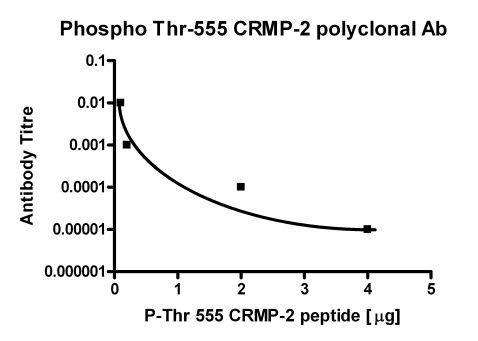


**A**

***Characterisation of anti-Phospho-Thr555 CRMP-2 antibody****.* ELISA determining phospho-Thr555 CRMP-2 peptide reactivity with the affinity purified polyclonal antibody (A). Immunoreactivity occurs at 1:10,000 dilution of the anti-Phospho-CRMP2 antibody with the phospho-CRMP2 peptide. Tg2576 brain lysates react with the anti-phospho-CRMP2 antibody by western blot (B). A ~62 kDa band appears with incubation of anti-phospho-CRMP2 antibody alone and this immunoreactivity can be blocked by pre-incubation of the antibody with the phosphor-Thr555 CRMP2 peptide. Pre-incubation with the same CRMP2 peptide without phosphorylation produces the same 62 kDa band. Incubation with the pre-bleed antiserum shows no reactivity with the 62 kDa band (B). The 62 kDa band was then analysed by mass spectrometry and confirmed as phosphorylated CRMP2.
