# Supplementary figures and images for: Interleukin-18 alters protein expressions of neurodegenerative diseases-linked proteins in human SH-SY5Y neuron-like cells
Source: Front Cell Neurosci. 2014 Aug 7;8:214. doi: 10.3389/fncel.2014.00214 (PMC4124869; doi:10.3389/fncel.2014.00214)

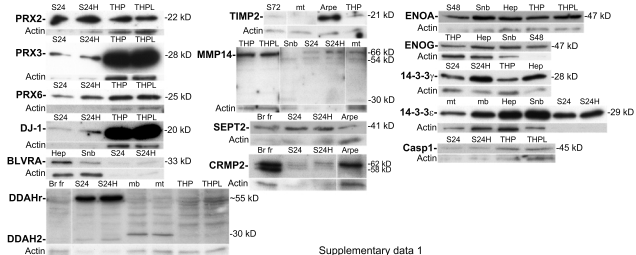

Supplement: Supplementary file 1 [file DataSheet1.ZIP › Supplementary Data 1.PDF]
